# Supplementary material for: Genomic Markers of CDK 4/6 Inhibitor Resistance in Hormone Receptor Positive Metastatic Breast Cancer
Source: Cancers (Basel). 2022 Jun 28;14(13):3159. doi: 10.3390/cancers14133159 (PMC9264913; doi:10.3390/cancers14133159)
Supplement: Supplementary file 1 [file cancers-14-03159-s001.zip › cancers-1774765-supplementary.pdf]

A

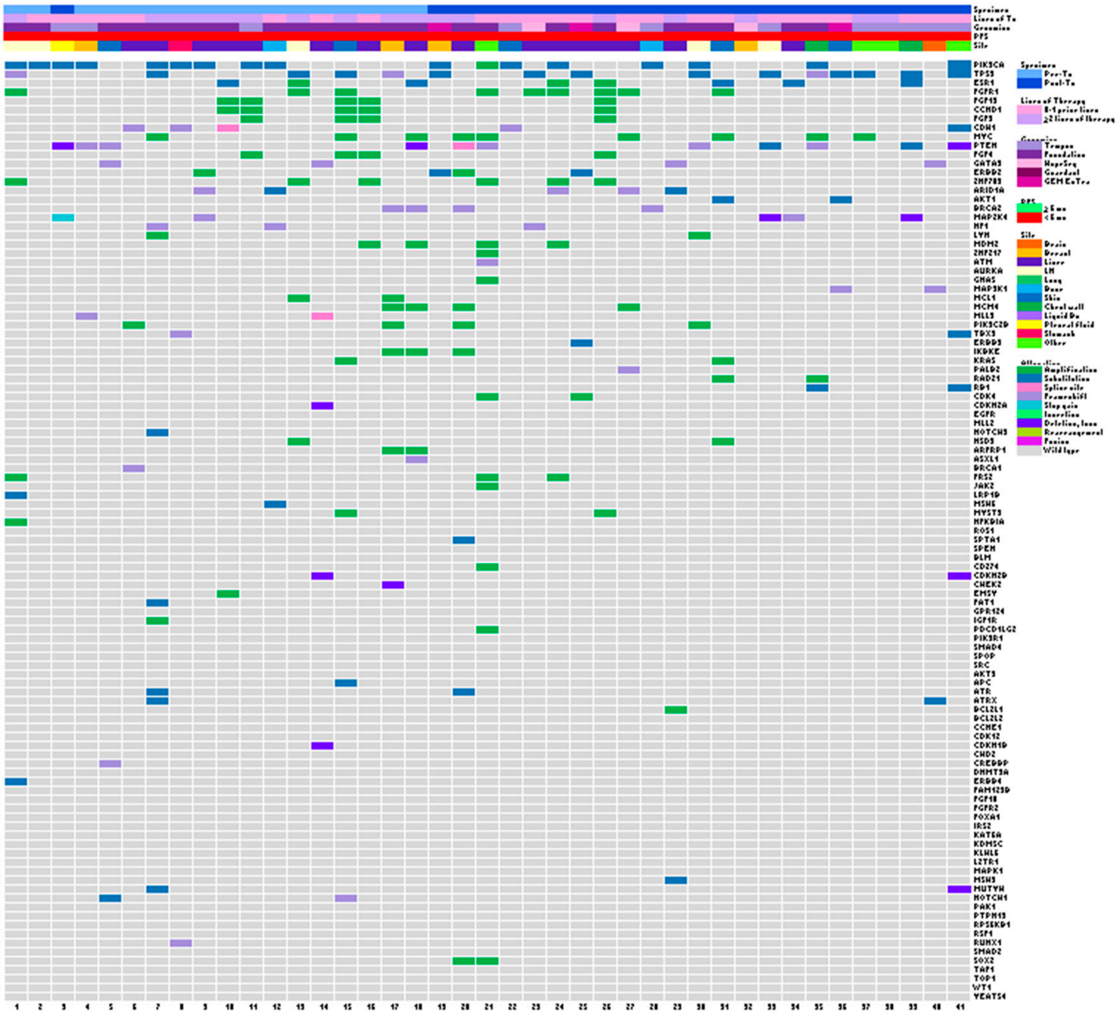

B

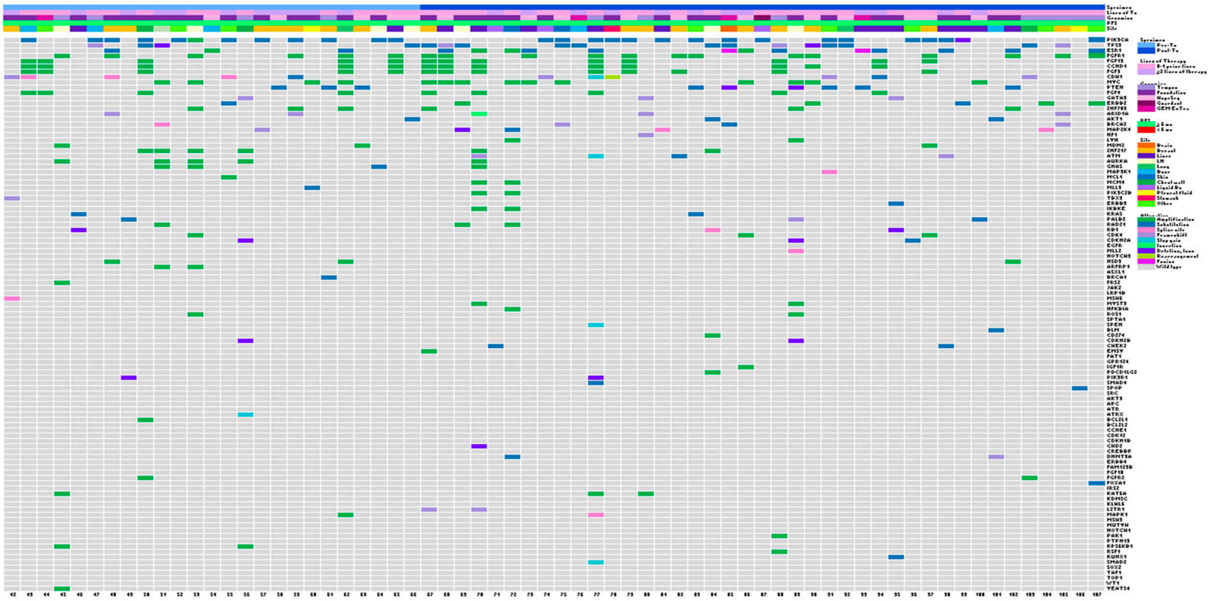

C

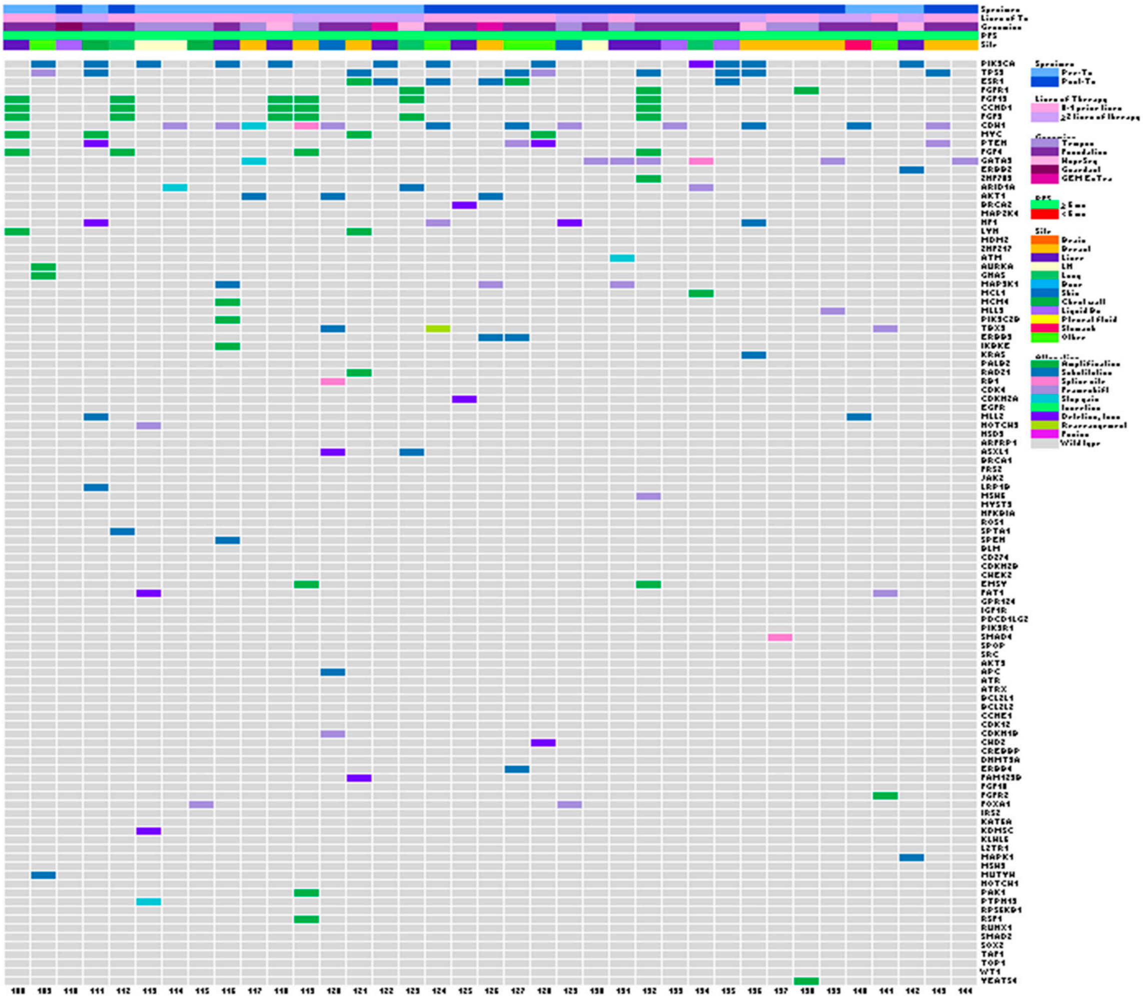

Figure S1. Tile plots of genomic alterations of each patient's tumor specimen: A) early progression (PFS < 6 months; N=41); B) intermediate progression (PFS 6-24 months for 0-1 lines; 6-9 months for 2+ lines; N=66); C) late progression (PFS >24 months for 0-1 lines; PFS >9 months for 2+ lines; N=37).

A

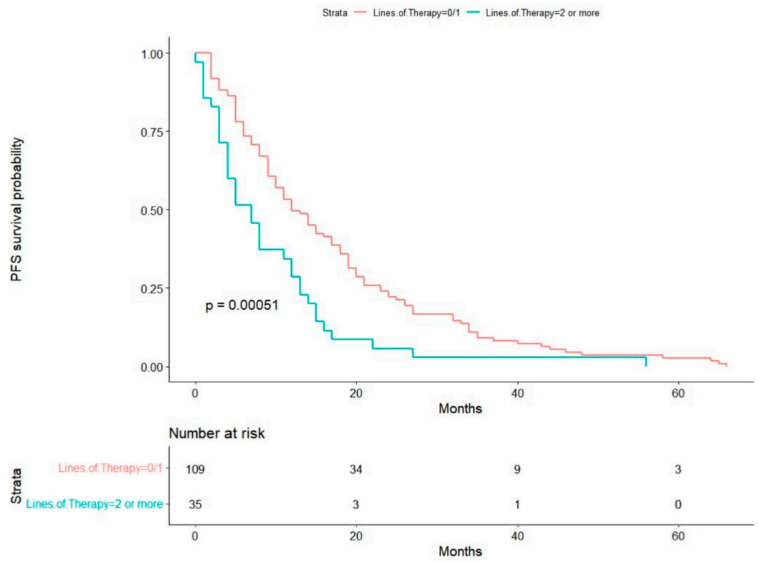

B

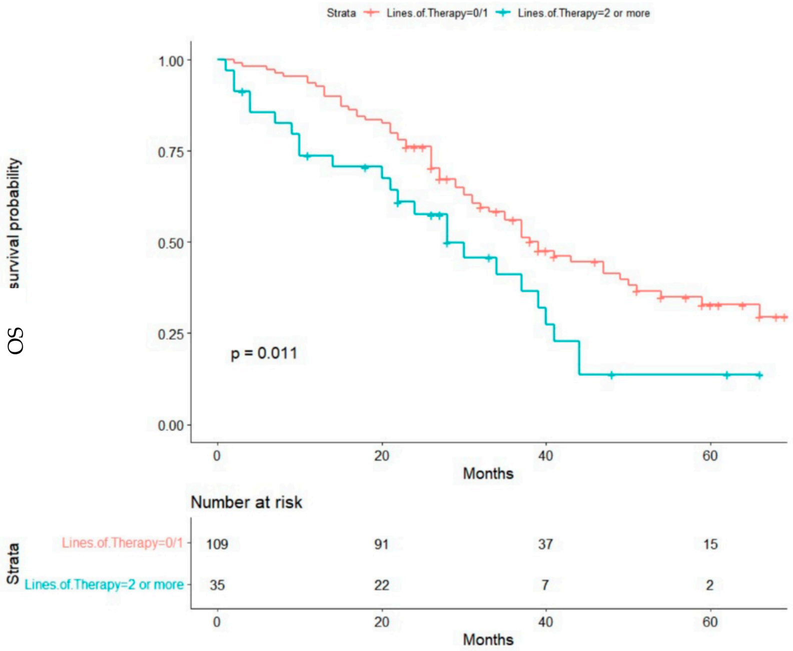

Figure S2. Kaplan-Meier survival analysis stratified by prior lines of chemotherapy: A) Median PFS for 0-1 prior lines (N=109) was 12 months (95% CI 10, 17) and for ≥ 2 lines (N=35) was 7 months (95% CI 4, 12); B) Median OS for 0-1 prior lines (N=109) was 39 months (95% CI 33, 51) and for ≥ 2 lines (N=35) was 28 months (95% CI 21, 41). Date of last follow up was used.

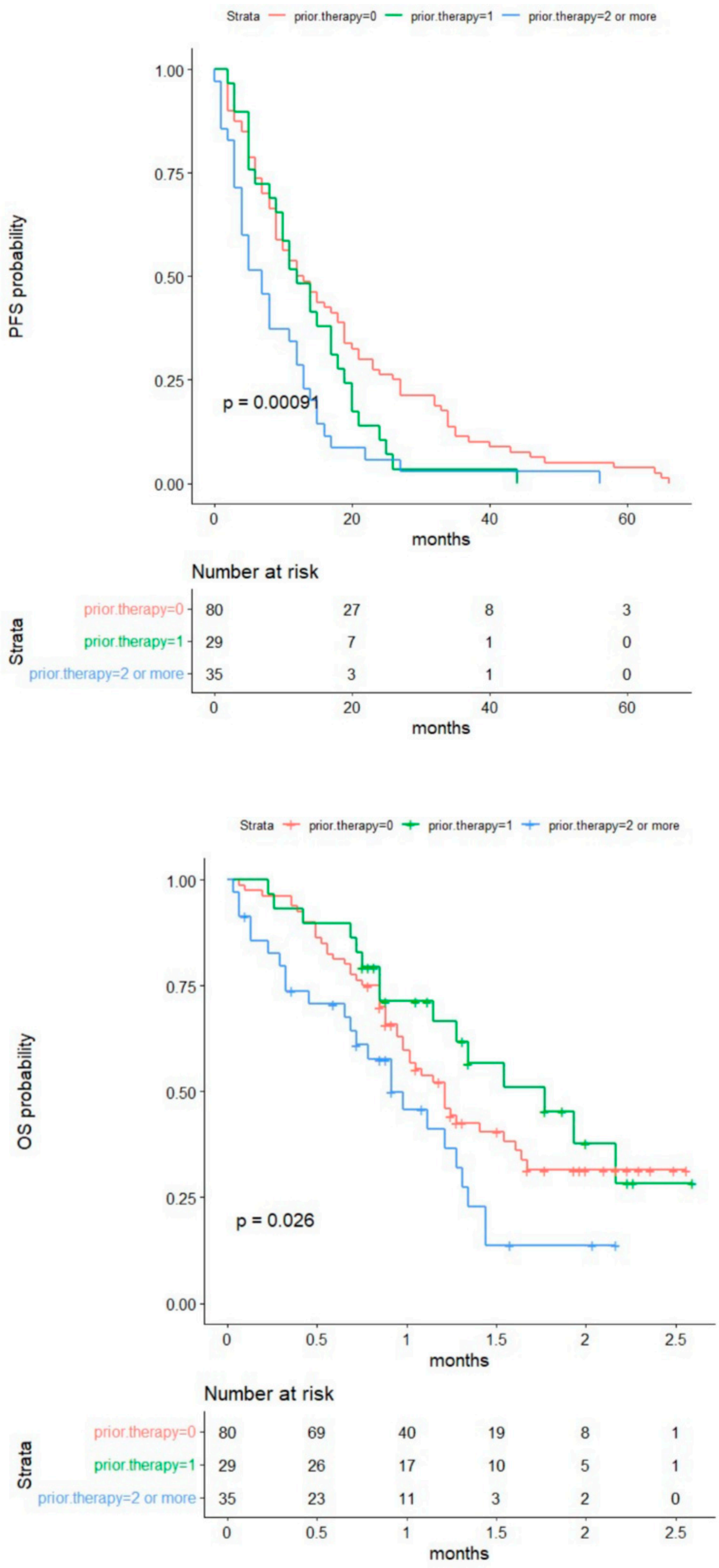

Figure S3. Kaplan-Meier survival analysis stratified by 0, 1, and ≥ 2 prior lines of chemotherapy: A) Median PFS for 0 prior lines (N=80, red line) was 12.5 months (95% CI 9—19); median PFS for 1 prior line (N=29, green line) was 12 months (95% CI 12—18);and median PFS for ≥ 2 lines (N=35, blue line) was 7 months (95% CI 4—12); B) Median OS for 0 prior lines (N=80, red line) was 37 months (95% CI 30—50);median PFS for 1 prior line (N=29, green line) was 54 months (95% CI 29—NA); and median PFS for ≥ 2 lines (N=35) was 28 months (95% CI 21—41).

Table S1. Tumor genomic analysis of patients with 0 or 1 line of prior therapy (n=109) and mutations that are present in $\geq$2 patients showed association with impaired PFS.

Table S2. RNA analysis for patients with 0 or 1 line of prior therapy (n=24) patients with 0-1 prior lines of therapy. 11,426 protein-coding genes were studied, and FDR was calculated to adjust for multiple comparisons. The 56 genes in the heatmap were selected with HR>4 or <0.25 with FDR <0.15. Unsupervised hierarchical clustering showed 3 clusters with distinct gene expression pattern. The 3 clusters are characterized by early, intermediate, and late PFS with the median PFS of 4.5 (n=4 range), 8.5 (n=12 range) and 29 (n=8, range) months, respectively. Statistical analysis was conducted in R (R-3.6.3, R Core Team).
